# Re-emergence of enterovirus D68 in Europe after easing the COVID-19 lockdown, September 2021

**DOI:** 10.2807/1560-7917.ES.2021.26.45.2100998

**Published:** 2021-11-11

**Authors:** Kimberley SM Benschop, Jan Albert, Andres Anton, Cristina Andrés, Maitane Aranzamendi, Brynja Armannsdóttir, Jean-Luc Bailly, Fausto Baldanti, Guðrún Erna Baldvinsdóttir, Stuart Beard, Natasa Berginc, Sindy Böttcher, Soile Blomqvist, Laura Bubba, Cristina Calvo, Maria Cabrerizo, Annalisa Cavallero, Cristina Celma, Ferruccio Ceriotti, Inês Costa, Simon Cottrell, Margarita del Cuerpo, Jonathan Dean, Jennifer L Dembinski, Sabine Diedrich, Javier Diez-Domingo, DagnyHaug Dorenberg, Erwin Duizer, Robert Dyrdak, Diana Fanti, Agnes Farkas, Susan Feeney, Jacky Flipse, Cillian De Gascun, Cristina Galli, Irina Georgieva, Laura Gifford, Raquel Guiomar, Mario Hönemann, Niina Ikonen, Marion Jeannoël, Laurence Josset, Kathrin Keeren, F Xavier López-Labrador, Melanie Maier, James McKenna, Adam Meijer, Beatriz Mengual-Chuliá, Sofie E Midgley, Audrey Mirand, Milagrosa Montes, Catherine Moore, Ursula Morley, Jean-Luc Murk, Lubomira Nikolaeva-Glomb, Sanela Numanovic, Massimo Oggioni, Paula Palminha, Elena Pariani, Laura Pellegrinelli, Antonio Piralla, Corinna Pietsch, Luis Piñeiro, Núria Rabella, Petra Rainetova, Sara Colonia Uceda Renteria, María P Romero, Marijke Reynders, Lieuwe Roorda, Carita Savolainen-Kopra, Isabelle Schuffenecker, Aysa Soynova, Caroline MA Swanink, Tina Ursic, Jaco J Verweij, Jorgina Vila, Tytti Vuorinen, Peter Simmonds, Thea K Fischer, Heli Harvala

**Affiliations:** 1 Center for Infectious Disease Control, National Institute for Public Health and the Environment, Bilthoven, the Netherlands; 2 Department of Clinical Microbiology, Karolinska University Hospital, Stockholm, Sweden; 3 Department of Microbiology, Tumor and Cell Biology, Karolinska Institutet, Stockholm, Sweden; 4 Respiratory Virus Unit, Microbiology Department, Vall d’Hebron Institut de Recerca (VHIR), Vall d’Hebron Barcelona Hospital Campus, Barcelona, Spain; 5 Microbiology Department, Donostia University Hospital and Biodonostia Health Research Institute, San Sebastián, Spain; 6 Landspitali-National University Hospital, Reykjavik, Iceland; 7 CHU Clermont-Ferrand, National Reference Centre for enteroviruses and parechoviruses – Associated laboratory, Clermont-Ferrand, France; 8 Université d’Auvergne, LMGE UMR CNRS 6023, Equipe EPIE - Epidémiologie et physiopathologie des infections à entérovirus, Faculté de Médecine, Clermont-Ferrand, France; 9 Microbiology and Virology Department, Fondazione IRCCS Policlinico San Matteo, Italy; 10 Department of Clinical Surgical Diagnostic and Pediatric Sciences, Università degli Studi di Pavia, Pavia, Italy; 11 UK Health Security Agency, Colindale, United Kingdom; 12 National laboratory of health, environment and food, Laboratory for public health virology, Ljubljana, Slovenia; 13 National Reference Center for Poliomyelitis and Enteroviruses, Robert-Koch Institute, Berlin, Germany; 14 National Institute for Health and Welfare, Helsinki, Finland; 15 Department of Biomedical Sciences of Health, University of Milan, Milan, Italy; 16 Hospital Universitario La Paz, Madrid, Spain; 17 National Centre for Microbiology, Instituto de Salud Carlos III, Enterovirus and Viral Gastroenteritis Unit/Polio National Lab, Madrid, Spain; 18 Laboratory of Microbiology, ASST Monza, San Gerardo Hospital, Monza (MB), Italy; 19 Virology Unit, Division of Clinical Laboratory, Fondazione IRCCS Ca' Granda Ospedale Maggiore Policlinico, Milan, Italy; 20 National Institute of Health (INSA), Lisbon, Portugal; 21 Public Health Wales, Cardiff, United Kingdom; 22 Microbiology Department Hospital Universitari de la Santa Creu i Sant Pau, Universitat Autònoma de Barcelona, Barcelona, Spain; 23 National Virus Reference Laboratory, University College Dublin, Dublin, Ireland; 24 Department of Virology, Norwegian Institute of Public Health, Oslo, Norway; 25 Center for Public Health Research (FISABIO-Public Health), Generalitat Valenciana, Valencia, Spain; 26 Chemical-clinical and Microbiological Analyses, ASST Grande Ospedale Metropolitano Niguarda, Milan, Italy; 27 National Public Health Center, Budapest, Hungary; 28 Regional Virus Laboratory, Belfast Health and Social Care Trust (BHSCT, Royal Victoria Hospital, Belfast, United Kingdom; 29 Laboratory for Medical Microbiology and Immunology, Rijnstate, Velp, the Netherlands; 30 National Reference Laboratory for Enteroviruses, National Center of Infectious and Parasitic Diseases, Sofia, Bulgaria; 31 Institute of Medical Microbiology and Virology, University of Leipzig, Leipzig, Germany; 32 National Reference Center for Enteroviruses and Parechoviruses, Institut des Agents Infectieux, Hospices Civils de Lyon, Lyon, France; 33 Secretary of the commission for Polio Eradication in Germany, Robert-Koch Institute, Berlin, Germany; 34 CIBERESP, Instituto de Salud Carlos III, Madrid, Spain; 35 The Danish WHO National Reference Laboratory for Poliovirus, Statens Serum Institut, Copenhagen, Denmark; 36 Elisabeth Tweesteden Hospital, Tilburg, the Netherlands; 37 National Institute of Public Health, Prague, Czech Republic; 38 Laboratory Medicine, Molecular Microbiology, AZ St.Jan Brugge-Oostende AV, Bruges, Belgium; 39 Maasstad Hospital, Rotterdam, the Netherlands; 40 Institute of Microbiology and Immunology, Faculty of Medicine, University of Ljubljana, Ljubljana, Slovenia; 41 Pediatric Hospitalization Unit, Department of Pediatrics, Hospital Universitari Vall d’Hebron, Vall d’Hebron Barcelona Hospital Campus, Barcelona, Spain; 42 Clinical Microbiology, Turku University Hospital and Institute of Biomedicine, University of Turku, Turku, Finland; 43 University of Oxford, Oxford, United Kingdom; 44 University of Sothern Denmark, Odense, Denmark; 45 Nordsjaellands Hospital, Hillerod, Denmark; 46 NHS Blood and Transplant, Microbiology Services, Colindale, United Kingdom; 47 University College London (UCL), Department of infection and Immunity, London, United Kingdom

**Keywords:** enterovirus D68, respiratory infection, enterovirus, typing, acute flaccid myelitis, surveillance

## Abstract

We report a rapid increase in enterovirus D68 (EV-D68) infections, with 139 cases reported from eight European countries between 31 July and 14 October 2021. This upsurge is in line with the seasonality of EV-D68 and was presumably stimulated by the widespread reopening after COVID-19 lockdown. Most cases were identified in September, but more are to be expected in the coming months. Reinforcement of clinical awareness, diagnostic capacities and surveillance of EV-D68 is urgently needed in Europe.

Coordination of case reporting by the European Non-Polio Enterovirus Network (ENPEN) found evidence for increasing numbers of enterovirus D68 (EV-D68) infections in Europe in September 2021. This prompted the consortium to send an alert email to member laboratories, requesting an urgent EV-D68 investigation. Here, we report the virological and clinical characteristics of 139 EV-D68 cases identified in eight European countries between 31 July and 14 October 2021.

## Enterovirus D68 detection and typing

A total of 36 institutions including 19 public health and 17 hospital laboratories from 18 European countries responded to the ENPEN alert (Table 1). 

**Table 1:** Details for 36 institutions reporting to this study and number of EV-D68 cases identified via their enterovirus and EV-D68 surveillance systems, 18 European countries, 1 January–14 October 2021 (n = 139 cases)

Country	Code	Type of laboratory	Enterovirus surveillance	Samples subjected to EV-D68 testing	Use of EV-D68-specific PCR	Molecular EV typing	Number of EV-D68 cases
Belgium	BE-01	Hospital	NA	All clinical respiratory samples	Yes	No	14
Bulgaria	BG-01	Public Health	AFP and EVCS	All EV-positive samples	No	No	0
Czechia	CZ-01	Public Health	EVCS	Not currently in place	No	No	0
Germany	DE-01	Public Health	EVCS	Limited respiratory samples^a^	No	In-house	0
	DE-02	Academic Hospital	ILI/ARI	EV-positive samples	No	In-house	0
Denmark	DK-01	Public Health	ILI/ARI and EVCS	All EV-positive samples	No	In-house	0
Spain	ES-01	Academic Hospital	EVCS	All EV-positive samples	No	In-house	9
	ES-02	Academic Hospital	EVCS	All EV-positive samples	No	In-house	1
	ES-03	Academic Hospital	NA	All EV-positive samples	No	ES-05	0
	ES-04	Academic Hospital	NA	NK	NK	NK	0
	ES-05	Public Health	AFP and EVCS	All EV-positive samples	No	In-house	0
	ES-06	Public Health	ILI/ARI	EV-positive repiratory samples	No	In-house	0
Finland	FI-01	Academic Hospital	NA	EV-positive samples	No	In-house	0
	FI-02	Public Health	ILI/ARI	EV-positive repiratory samples	Yes	In-house	0
France	FR-01	Academic Hospital	EVCS	All EV-positive samples and all HRV-EV positive respiratory samples	No	In-house	14
	FR-02	Public Health	EVCS	All EV-positive samples	Yes	In-house	6
Hungary	HU-01	Public Health	AFP and EVCS	All EV-positive samples	No	In-house	0
Ireland	IE-01	Public Health	EVCS	Proportion of EV-positive respiratory samples	Yes	In-house	1
Iceland	IS-01	Public Health	EVCS	All EV-positive samples	Yes	In-house	0
Italy	IT-01	Public Health	ILI/ARI and AFP	All EV-positive samples	Yes	In-house	1
	IT-02	Academic Hospital	ILI/ARI	All clinical respiratory samples	Yes	In-house	0
	IT-03	Academic Hospital	NA	All clinical respiratory samples	No	IT-01/IT-02	0
	IT-04	Hospital	NA	All clinical respiratory samples	No	IT-01/IT-02	0
	IT-05	Hospital	NA	All clinical respiratory samples	No	IT-01/IT-02	0
The Netherlands	NL-01	Hospital	NA	All clinical respiratory samples	No	In-house	1
	NL-02-A	Public Health	EVCS	All EV-positive samples	No	In-house	1
	NL-02-B		ILI/ARI	All clinical respiratory samples	Yes	In-house	0
	NL-03	Hospital	NA	All clinical respiratory samples	No	In-house	0
	NL-04	Hospital	NA	All clinical respiratory samples	No	In-house	0
Norway	NO-01	Public Health	AFP and EVCS	All EV-positive samples	Yes	In-house	0
Portugal	PT-01	Public Health	NA	All clinical respiratory samples	No	No	0
Sweden	SE-01	Academic Hospital	NA	All clinical respiratory and EV-positive samples	Yes	In-house^b^	2
Slovenia	SI-01	Public Health	ILI/ARI and AFP	All EV-positive samples	No	In-house	0
	SI-02	Academic Hospital	NA	Mostly respiratory samples	Yes	In-house	0
England, UK	UK-01	Public Health	EVCS	All EV-positive samples	Yes	In-house	7
Wales, UK	UK-02	Public Health	ILI/ARI and EVCS	All EV-positive samples	Yes	In-house	82
Belfast, UK	UK-03	Hospital	NA	Any samples if clinically indicated	Yes	In-house	0

AFM: acute flaccid myelitis surveillance; AFP: acute flaccid paralysis surveillance; EV: enterovirus; EVCS: EV clinical surveillance (i.e. EV-positive clinical specimens are subjected to typing); HRV: human rhinovirus; ILI/ARI: sentinel influenza surveillance focusing on influenza like illness and acute respiratory infection; NA: not applicable; NK: not known; UK: United Kingdom.

^a^ Only if respiratory sample has been taken from a case with acute flaccid paralysis.

^b^ EV-positive faecal and central nervous system samples referred for typing at public health agency.

We requested data on EV-D68 cases and detection methods. The 19 public health laboratories from 13 countries indicated that they would identify EV-D68 infections either via their EV surveillance (n = 14), surveillance focusing on influenza-like illness (ILI) and/or acute respiratory infection (ARI; n = 8), or via surveillance for acute flaccid paralysis (AFP) (n = 6). Two laboratories (in the Czech Republic and Germany) had not included respiratory samples in their EV surveillance. Data on screening and typing were supplied by 33 laboratories. All except one of the 14 hospital laboratory tested respiratory samples for EV-D68. The use of EV-D68-specific PCR was reported by 13 of 33 laboratories, and genetic characterisation by sequencing was applied in most laboratories (28/33, Table 2).

**Table 2:** Laboratory details for enterovirus detection and typing, 18 European countries, 1 January–14 October 2021 (n = 36 laboratories)

Country	Code	EV and screening methods	EV sequencing methods
Belgium	BE-01	Faeces: EV PCR on GI-TAC assay; respiratory samples: EV PCR and EV-D68 PCR on respiratory TAC assay; others: in-house EV PCR; CSF: FilmArray panel	NA
Bulgaria	BG-01	All samples: cell culture (A), and EV PCR (B)	NA
Czechia	CZ-01	Faeces: cell culture and EV PCR	NA
Germany	DE-01	Only faeces and CSF tested, respiratory (only AFP cases): EV PCR	Complete or partial VP1 region
	DE-02	All samples: EV-PCR and EV/HRV PCR	Partial VP1
Denmark	DK-01	All samples: EV PCR and HRV PCR	Partial VP1 and VP4-VP2 for EV, VP2 for RV
Spain	ES-01	All samples: HRV16 Allplex Respiratory Panel (Seegene) or RealCycle EV/hPeV detection (Progenie)	Partial VP1
	ES-02	Respiratory samples: Allplex Respiratory Panel; other samples: EV PCR	Partial VP1
	ES-03	All respiratory samples negative for other respiratory viruses, CSF and faeces from neurological or cutaneous illnesses	Partial VP1
	ES-04	No data	Partial VP1
	ES-05	All samples: EV PCR	Partial VP1
	ES-06	Respiratory samples: respiratory RT-PCR panel (EV/HRV in one channel)	EV-D68 typing PCR
Finland	FI-01	Respiratory samples: EV/HRV PCR], All samples: EV-PCR	Partial VP1
	FI-02	Respiratory samples: EV/HRV PCR and EV-D68 PCR	Complete VP1 and VP4-VP2
France	FR-01	Respiratory samples: EV/HRV PCR	Complete or partial VP1 and VP4-VP2
	FR-02-A	All samples: EV PCR and EV/HRV PCR	Complete or partial VP1 and VP4-VP2; complete or partial VP1 and VP4-VP2
	FR-02-B	Respiratory samples (< 5 years) or samples from severe cases (respiratory AFM): EV-D68 PCR	
Hungary	HU-01	All samples: EV PCR	5'-NTR, partial VP1
Ireland	IE-01	Respiratory samples: Luminex NxTAG Respiratory Panel (EV/HRV); respiratory samples with clinical indication: EV-D68 PCR; all other samples: EV PCR	Partial VP1
Iceland	IS-01	All samples: EV PCR and EV-D68 PCR	Partial VP1
Italy	IT-01	Respiratory samples: EV PCR and EV-D68 PCR	Partial VP1 and VP4-VP2
	IT-02	Respiratory samples: EV/HRV PCR and EV-D68 PCR	EV-D68 typing; HRV/EV on VP4/VP2 typing VP1 typing
	IT-03	Respiratory samples: EV PCR: Allplex Respiratory Panel	NA
	IT-04	Respiratory samples: EV PCR: Allplex Respiratory Panel	NA
	IT-05	Respiratory samples: EV PCR: Allplex Respiratory Panel	NA
The Netherlands	NL-01	All samples: EV PCR	Partial VP1
	NL-02-A	All samples: EV PCR	Partial VP1
	NL-02-B	Respiratory samples: EV PCR and HRV PCR and EV-D68 PCR	5’-NTR, EV-D68 VP1, partial VP1
	NL-03	All samples: EV PCR	Partial VP1
	NL-04	All samples: EV PCR	Partial VP1
Norway	NO-01	All samples: EV PCR and EV-D68 PCR	Partial VP1
Portugal	PT-01	Respiratory samples: EV PCR: Allplex Respiratory Panel	NA
Sweden	SE-01-A	Non-respiratory samples: EV PCR	Partial VP1 and VP4-VP2
	SE-01-B	Respiratory samples: Allplex Respiratory Panel and EV-D68 PCR	Partial VP1 and VP4-VP2
Slovenia	SI-01	Respiratory samples: EV PCR	Partial VP1
	SI-02	All samples: EV-D68 PCR	Partial VP1
England, UK	UK-01	All samples: EV PCR and EV-D68 PCR	Partial VP1
Wales, UK	UK-02	All respiratory samples/CNS/faeces: EV PCR and EV-D68 PCR	Partial VP1
Belfast, UK	UK-03	Respiratory samples with clinical indication; skin swab with clinical indication; all CSF samples; blood with clinical symptoms; EV PCR and EV-D68 PCR	Partial VP1 Colindale reference laboratory sequencing

AFM: acute flaccid myelitis; CSF: cerebrospinal fluid; EV: enterovirus; GI-TAC: gastrointestinal Taqman array card; hPeV: human parechovirus; HRV: human rhinovirus; NA: not applicable; NTR: non-translated region; UK: United Kingdom; VP: viral protein.

References for the methods presented here are listed in the Supplement.

## Distribution of enterovirus D68 cases in Europe

A total of 139 EV-D68 cases were identified between 31 July and 14 October 2021 by 12 laboratories in eight countries (Table 1). Most EV-D68-positive samples were collected in September (99/139, 71%, Figure). Screening and typing of samples collected in October is ongoing.

**Figure:**
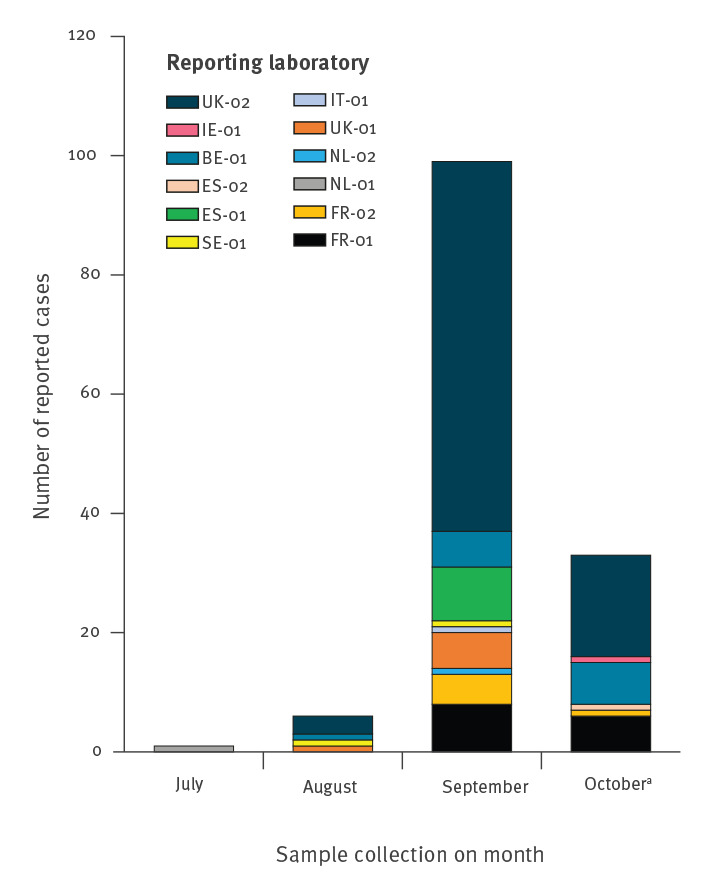
EV-D68 detection in Europe, 1 July–14 October 2021 (n = 139)

BE: Belgium; ES: Spain; EV: enterovirus; FR: France; IE: Ireland; IT: Italy; NL: the Netherlands; SE: Sweden; UK: United Kingdom.

^a^ Data incomplete based on samples collected and reported as EV-D68 by 14 October 2021.

## Number of EV-D68 infections is increasing in Europe

Denominator data were available from 24 laboratories reporting 66 of 139 EV-D68 cases. Since the beginning of 2021, these laboratories identified 1,964 EV-positive samples by screening 56,401 samples by EV PCR (some also detecting and hence including rhinoviruses in these reports). While the number of samples tested monthly remained unchanged (on average 6,113 samples screened monthly between January and July, 5,170 in August and 6,353 in September), an increase in the positivity rate was noted (overall 2.5% in January through July, 4.5% in August and 8.2% in September). A total of 967 samples were typed as EV and 36 as EV-D68. The proportion of samples identified as EV-D68 increased from 0.2% in January through July (1/409) and 0.9% in August (2/208) to 14% in September (33/236; p < 0.0001 by chi-squared test). 

An additional 30 EV-D68-positive samples were identified by screening of 8,243 respiratory samples by EV-D68-specific PCR in 13 laboratories. Of these, none was identified before August despite screening of 5,088 samples since January (monthly average: 727 samples). Three EV-D68 positive samples were identified in August (3/739; 0.4%) and 27 in September and October (27/1,289; 2%) demonstrating a recent but significant increase in the EV-D68 positivity rate (p < 0.0001 by chi-squared test).

## Clinical characteristics of enterovirus D68 cases

Demographic information was collected for all reported EV-D68 cases (Table 3). Most were males (88/139; 63%) and younger than 5 years (120/139; 86%), with a median age of 3 years (range: newborns to 72 years). Clinical symptoms were reported for 120 cases, with most exhibiting respiratory symptoms (n = 116; 97%). Although five cases had neurological symptoms, none was diagnosed with AFP or acute flaccid myelitis (AFM). Thirty of 49 cases with data on hospitalisation were hospitalised. Pre-existing conditions were reported for 20 of 45 cases with available information, predominantly in older age groups (2/14 < 2 years, 18/31 > 2 years). Viral co-infection was reported for 16 cases; the most common co-detection was rhinovirus (n = 9). To date, genotyping of 20 EV-D68 positive samples has shown all strains to be genotype B3 (data not shown).

**Table 3:** Demographic and clinical characteristics of individuals with a laboratory-confirmed EV-D68 infection, eight European countries, 1 September–14 October 2021 (n = 139)

	Number of cases	Proportion of cases
**Age group**		
0–3 months	7	5%
4–12 months	15	11%
13–24 months	22	16%
2–5 years	76	55%
6–15 years	9	6%
16–25 years	2	1%
26–45 years	2	1%
> 45 years	6	4%
**Sex**		
Female	51	37%
Male	88	63%
**Symptoms (data reported for)**		
Any symptom reported (n = 121)	120	99%
Fever (n = 111)	49	44%
Enteric symptoms (n = 120)	4	3%
Respiratory symptoms (n = 120)	116	97%
Neurological symptoms^a^ (n = 111)	5	5%
**Clinical information (data reported for)**		
Hospitalised (n = 49)	30	
Pre-existing condition^b^ (n = 45)	20	
**Co-infections (data reported for)**		
Any co-infection reported (n = 43)	16	
Adenovirus	4	
Rhinovirus	6	
Human metapneumovirus	1	
Adenovirus and rhinovirus	1	
Adenovirus and bocavirus	1	
Rhinovirus and SARS-CoV-2	1	
PIV4 and CMV	1	
Rhinovirus, bocavirus and PIV3	1	

CMV: cytomegalovirus; EV: enterovirus; PIV: parainfluenza virus; SARS-CoV-2: severe acute respiratory syndrome coronavirus 2.

^a^ Reported neurological symptoms included headache, dizziness and agitation.

^b^ Reported pre-existing conditions were asthma, chronic obstructive pulmonary disease, sleep apnoea and immunosuppression.

## Discussion

Enterovirus D68 (EV-D68) infections have been linked to AFP/AFM since a large outbreak associated with respiratory and neurological symptoms in children was described in North America in 2014 [1,2]. Although regular EV-D68 upsurges have been reported in Europe since 2010 [2-7], they largely ceased during the coronavirus disease (COVID-19) pandemic. Here we report EV-D68 circulation across Europe for the first time following the COVID-19 pandemic, with case numbers already exceeding what was reported during the most recent EV-D68 upsurge in 2019 [7].

Although EV-D68 circulation in Europe has largely followed a biennial epidemic pattern confined to the autumn season of even-numbered years, the autumn of 2019 showed an unexpected upsurge of EV-D68 infections leading to 93 reported cases, two with AFM, in five European countries [7]. EV-D68 has largely been detected through ILI/ARI sentinel surveillance because of its respiratory signature and in EV surveillance systems which have included respiratory samples since the occurrence of the first large outbreak of EV-D68 in Europe and North America where these samples were recommended [8,9]. Several institutes have additionally included EV-D68-specific PCR for their respiratory surveillance.

The timing of this increase in the number of EV-D68 infections is consistent with the known seasonality of EV, with numbers usually peaking in September and October [10]. However, this marked upsurge is likely to have been further precipitated by the widespread relaxation of COVID-19 mitigation measures such as travel restrictions, school closures, use of face masks and physical distancing. The findings are indeed consistent with the widespread resurgence of other community-transmitted respiratory infections, whose circulation in most of Europe had until recently largely ceased [11,12]. The interruption in the transmission of respiratory and enteric viruses, including EV-D68, has probably created large cohorts of susceptible young children without prior exposure or immunity to any such virus, potentially creating the conditions for largescale outbreaks of severe respiratory disease in this age group this winter.

As circulation of EV diminished during the lockdown, many of the surveillance systems not related to SARS-CoV-2 were temporarily discontinued or received fewer specimens because testing facilities prioritised SARS-CoV-2. Although most study participants have now re-started active investigations via established surveillance systems, it is also important to consider the minimum number of samples needed for effective surveillance. Nonetheless, laboratories in 16 countries use screening or surveillance systems that enable detection of EV-D68 infection through typing of EV-positive samples. At the time of our previous survey on the EV laboratory and surveillance capacity in Europe in 2016, only 11 countries had introduced or modified their existing surveillance systems to enable EV-D68 detection [8]. This clearly demonstrates increased capacity for detection and awareness of EV-D68 across Europe, probably a sign of the strength of the continued collaboration established through ENPEN [9,13-16]. Notably, the zero-reporting noted by two countries in this study was due to the exclusion of respiratory samples from their EV surveillance, an important reminder that a respiratory sample is needed for the detection of EV-D68 (even in cases of AFM) as the virus is only rarely detected in faecal or cerebrospinal fluid samples [9].

Our data clearly demonstrate that EV-D68 is now circulating in Europe, mostly affecting children or those with underlying conditions. Most paediatric cases presented with respiratory symptoms. Although no AFM cases were reported, we should be alert to the possibility of EV-D68-associated AFM cases occurring in the coming months following the rise in EV-D68 cases most evident in Wales, Belgium, France and Spain. The same trends were noted during the North American outbreak in 2014 where the majority of the initial cases were associated with respiratory diseases and neurological cases were only observed with a delay of a few weeks [1]. 

We recommend sequence analysis of EV-D68 to determine the relatedness of viruses circulating in Europe, and their potential link to a novel B3 subclade reported in 2019 [7]. The existing surveillance systems as well as laboratory and clinical networks relating to EV-D68 should be activated as this infection can have severe consequences [17-20].

## Conclusion

Re-emergence of EV-D68, and its known association with several neurological infections, is a reminder that the surveillance for EV infections is important. This study shows that EV-D68 cases can be identified through a combination of ILI/ARI sentinel surveillance and EV surveillance expanded to include respiratory samples. It calls for continued careful monitoring and vigilant testing of respiratory samples. 

## Supplementary Data

244000 Supplement
