# Supplementary material for: Re-emergence of enterovirus D68 in Europe after easing the COVID-19 lockdown, September 2021
Source: Euro Surveill. 2021 Nov 11;26(45):2100998. doi: 10.2807/1560-7917.ES.2021.26.45.2100998 (PMC8646978; doi:10.2807/1560-7917.ES.2021.26.45.2100998)
Supplement: Supplement [file 21-00998_HARVALA_SupplementaryReferences.pdf]

Supplementary References for Table 2 in the main manuscript

This supplementary material is hosted by *Eurosurveillance* as supporting information alongside the article "Re-emergence of enterovirus D68 in Europe after easing the COVID-19 lockdown, September 2021", on behalf of the authors, who remain responsible for the accuracy and appropriateness of the content. The same standards for ethics, copyright, attributions and permissions as for the article apply. Supplements are not edited by *Eurosurveillance* and the journal is not responsible for the maintenance of any links or email addresses provided therein.

TABLE 2. Laboratory details for enterovirus detection and typing, 18 European countries, 1 January–14 October 2021 (n = 36 laboratories). References for the methods presented here are provided in the supplementary file.

Country	Code	EV and screening methods	EV sequencing methods
Belgium	BE-01	Faeces: EV PCR on GI-TAC assay; respiratory samples: EV PCR and EV-D68 PCR on respiratory TAC assay; others: in-house EV PCR; CSF: FilmArray panel	NA
Bulgaria	BG-01	All samples: cell culture (A), and EV PCR (B)	NA
Czechia	CZ-01	Faeces: cell culture and EV PCR	NA
Gemany	DE-01	Only faeces and CSF tested, respiratory (only AFP cases): EV PCR[1]	Complete or partial VP1 region[1]
	DE-02	All samples: EV-PCR and EV/HRV PCR	Partial VP1[2]
Denmark	DK-01	All samples: EV PCR and HRV PCR[3]	Partial VP1 and VP4-VP2 for EV, VP2 for RV[2,4,5]
Spain	ES-01	All samples: HRV16 Allplex Respiratory Panel (Seegene) or RealCycle EV/hPeV detection (Progenie)	Partial VP1[2]
	ES-02	Respiratory samples: Allplex Respiratory Panel; other samples: EV PCR	Partial VP1[6,7]
	ES-03	All respiratory samples negative for other respiratory viruses, CSF and faeces from neurological or cutaneous illnesses	Partial VP1[6,7]
	ES-04	No data	Partial VP1[6,7]
	ES-05	All samples: EV PCR	Partial VP1[6,7]
	ES-06	Respiratory samples: respiratory RT-PCR panel (EV/HRV in one channel)[8]	EV-D68 typing PCR[8]
Finland	FI-01	Respiratory samples: EV/HRV PCR], All samples: EV-PCR	Partial VP1[2]
	FI-02	Respiratory samples: EV/HRV PCR and EV-D68 PCR	Complete VP1 and VP4-VP2[2]
France	FR-01	Respiratory samples: EV/HRV PCR[9]	Complete or partial VP1 and VP4-VP2[2,9–13]
	FR-02-A	All samples: EV PCR and EV/HRV PCR	Complete or partial VP1 and VP4-VP2; complete or partial VP1 and VP4-VP2[2,9,11–14]
	FR-02-B	Respiratory samples (< 5 years) or samples from severe cases (respiratory AFM): EV-D68 PCR	
Hungary	HU-01	All samples: EV PCR[15]	5-NTR, partial VP1[2]
Ireland	IE-01	Respiratory samples: Luminex NxTAG Respiratory Panel (EV/HRV); respiratory samples with clinical indication: EV-D68 PCR; all other samples: EV PCR[2]	Partial VP1[2]
Iceland	IS-01	All samples: EV PCR and EV-D68 PCR[16,17]	Partial VP1[18,19]
Italy	IT-01	Respiratory samples: EV PCR and EV-D68 PCR[20,21]	Partial VP1 and VP4-VP2[2,10,14,22,23]
	IT-02	Respiratory samples: EV/HRV PCR and EV-D68 PCR[21]	EV-D68 typing; HRV/EV on VP4/VP2 typing VP1 typing[2,10,14,22,23]

	IT-03	Respiratory samples: EV PCR: Allplex Respiratory Panel	NA
	IT-04	Respiratory samples: EV PCR: Allplex Respiratory Panel	NA
	IT-05	Respiratory samples: EV PCR: Allplex Respiratory Panel	NA
The Netherlands	NL-01	All samples: EV PCR[24]	Partial VP1[2]
	NL-02-A	All samples: EV PCR[24,25]	Partial VP1[2]
	NL-02-B	RespiratorySamples: EV PCR and HRV PCR and EV-D68 PCR adapted from[8]	5'NTR[26], EV-D68 VP1, partial VP1[2]
	NL-03	All samples: EV PCR[24]	Partial VP1[2]
	NL-04	All samples: EV PCR[24]	Partial VP1[2]
Norway	NO-01	All samples: EV PCR and EV-D68 PCR[27]	Partial VP1[2]
Portugal	PT-01	Respiratorysamples: EV PCR: Allplex Respiratory Panel	NA
Sweden	SE-01-A	Non-respiratory samples: EV PCR[28]	Partial VP1 and VP4-VP2[2,23]
	SE-01-B	Respiratory samples: Allplex Respiratory Panel and EV-D68 PCR[21]	Partial VP1 and VP4-VP2[2,23]
Slovenia	SI-01	Respiratory samples: EV PCR[16]	Partial VP1[2]
	SI-02	All samples: EV-D68 PCR	Partial VP1[2]
England, UK	UK-01	All samples: EV PCR and EV-D68 PCR	Partial VP1[2]
Wales, UK	UK-02	All respiratory samples/CNS/faeces: EV PCR and EV-D68 PCR	Partial VP1[2]
Belfast, UK	UK-03	Respiratory samples with clinical indication; skin swab with clinical indication; all CSF samples; blood with clinical symptoms; EV PCR and EV-D68 PCR	Partial VP1 Colindale reference laboratory sequencing

AFM: acute flaccid myelitis; CSF: cerebrospinal fluid; EV: enterovirus; GI-TAC: gastrointestinal Taqman array card; hPeV: human parechovirus; HRV: human rhinovirus; NA: not applicable; NTR: non-translated region; UK: United Kingdom; VP: viral protein.

1. Keeren K, Bottcher S, Diedrich S. Enterovirus Surveillance (EVSurv) in Germany. Microrganisms. 2021;9(10):2005.
2. Nix WA, Oberste MS, Pallansch MA. Sensitive, seminested PCR amplification of VP1 sequences for direct identification of all enterovirus serotypes from original clinical specimens. J Clin Microbiol. 2006;44(8):2698-704.
3. Midgley SE, Christiansen CB, Poulsen MW, Hansen CH, Fischer TK. Emergence of enterovirus D68 in Denmark, June 2014 to February 2015. Euro surveillance : bulletin Europeen sur les maladies transmissibles = European communicable disease bulletin. 2015;20(17).
4. Nasri D, Bouslama L, Omar S, Saoudin H, Bourlet T, Aouni M, et al. Typing of human enterovirus by partial sequencing of VP2. J Clin Microbiol. 2007;45(8):2370-9.
5. Bønnelykke K, Coleman AT, Evans MD, Thorsen J, Waage J, Vissing NH, et al. Cadherin-related Family Member 3 Genetics and Rhinovirus C Respiratory Illnesses. American journal of respiratory and critical care medicine. 2018;197(5):589-94.

6. Cabrerizo M, Echevarria JE, González I, de Miguel T, Trallero G. Molecular epidemiological study of HEV-B enteroviruses involved in the increase in meningitis cases occurred in Spain during 2006. J Med Virol. 2008;80(6):1018-24.
7. González-Sanz R, Taravillo I, Reina J, Navascués A, Moreno-Docón A, Aranzamendi M, et al. Enterovirus D68-associated respiratory and neurological illness in Spain, 2014-2018. Emerging microbes & infections. 2019;8(1):1438-44.
8. Poelman R, Schölvinck EH, Borger R, Niesters HG, van Leer-Buter C. The emergence of enterovirus D68 in a Dutch University Medical Center and the necessity for routinely screening for respiratory viruses. J Clin Virol. 2015;62:1-5.
9. Schuffenecker I, Mirand A, Josset L, Henquell C, Hecquet D, Pilorgé L, et al. Epidemiological and clinical characteristics of patients infected with enterovirus D68, France, July to December 2014. Euro surveillance : bulletin Europeen sur les maladies transmissibles = European communicable disease bulletin. 2016;21(19).
10. Mirand A, le Sage FV, Pereira B, Cohen R, Levy C, Archimbaud C, et al. Ambulatory Pediatric Surveillance of Hand, Foot and Mouth Disease as Signal of an Outbreak of Coxsackievirus A6 Infections, France, 2014-2015. Emerg Infect Dis. 2016;22(11):1884-93.
11. Linsuwanon P, Payungporn S, Samransamruajkit R, Posuwan N, Makkoch J, Theanboonlers A, et al. High prevalence of human rhinovirus C infection in Thai children with acute lower respiratory tract disease. J Infect. 2009;59(2):115-21.
12. Savolainen C, Mulders MN, Hovi T. Phylogenetic analysis of rhinovirus isolates collected during successive epidemic seasons. Virus Res. 2002;85(1):41-6.
13. Bal A, Pichon M, Picard C, Casalegno JS, Valette M, Schuffenecker I, et al. Quality control implementation for universal characterization of DNA and RNA viruses in clinical respiratory samples using single metagenomic next-generation sequencing workflow. BMC infectious diseases. 2018;18(1):537.
14. Mirand A, Henquell C, Archimbaud C, Chambon M, Charbonne F, Peigue-Lafeuille H, et al. Prospective identification of enteroviruses involved in meningitis in 2006 through direct genotyping in cerebrospinal fluid. J Clin Microbiol. 2008;46(1):87-96.
15. Diedrich S, Driesel G, Schreier E. Sequence comparison of echovirus type 30 isolates to other enteroviruses in the 5'noncoding region. J Med Virol. 1995;46(2):148-52.
16. Dierssen U, Rehren F, Henke-Gendo C, Harste G, Heim A. Rapid routine detection of enterovirus RNA in cerebrospinal fluid by a one-step real-time RT-PCR assay. J Clin Virol. 2008;42(1):58-64.
17. Bragstad K, Jakobsen K, Rojahn AE, Skram MK, Vainio K, Holberg-Petersen M, et al. High frequency of enterovirus D68 in children hospitalised with respiratory illness in Norway, autumn 2014. Influenza and other respiratory viruses. 2015;9(2):59-63.

18. Oberste MS, Maher K, Kilpatrick DR, Flemister MR, Brown BA, Pallansch MA. Typing of human enteroviruses by partial sequencing of VP1. J Clin Microbiol. 1999;37(5):1288-93.

19. Oberste MS, Maher K, Kilpatrick DR, Pallansch MA. Molecular evolution of the human enteroviruses: correlation of serotype with VP1 sequence and application to picornavirus classification. J Virol. 1999;73(3):1941-8.

20. Poelman R, Schuffenecker I, Van Leer-Buter C, Josset L, Niesters HG, Lina B. European surveillance for enterovirus D68 during the emerging North-American outbreak in 2014. J Clin Virol. 2015;71:1-9.

21. Piralla A, Girello A, Premoli M, Baldanti F. A new real-time reverse transcription-PCR assay for detection of human enterovirus 68 in respiratory samples. J Clin Microbiol. 2015;53(5):1725-6.

22. Piralla A, Girello A, Grignani M, Gozalo-Margüello M, Marchi A, Marseglia G, et al. Phylogenetic characterization of enterovirus 68 strains in patients with respiratory syndromes in Italy. J Med Virol. 2014;86(9):1590-3.

23. Wisdom A, Leitch EC, Gaunt E, Harvala H, Simmonds P. Screening respiratory samples for detection of human rhinoviruses (HRVs) and enteroviruses: comprehensive VP4-VP2 typing reveals high incidence and genetic diversity of HRV species C. J Clin Microbiol. 2009;47(12):3958-67.

24. Benschop K, Molenkamp R, van der Ham A, Wolthers K, Beld M. Rapid detection of human parechoviruses in clinical samples by real-time PCR. J Clin Virol. 2008;41(2):69-74.

25. Jaramillo-Gutierrez G, Benschop KS, Claas EC, de Jong AS, van Loon AM, Pas SD, et al. September through October 2010 multi-centre study in the Netherlands examining laboratory ability to detect enterovirus 68, an emerging respiratory pathogen. J Virol Methods. 2013;190(1-2):53-62.

26. Meijer A, van der Sanden S, Snijders BE, Jaramillo-Gutierrez G, Bont L, van der Ent CK, et al. Emergence and epidemic occurrence of enterovirus 68 respiratory infections in The Netherlands in 2010. Virology. 2012;423(1):49-57.

27. Hayes A, Nguyen D, Andersson M, Antón A, Bailly JL, Beard S, et al. A European multicentre evaluation of detection and typing methods for human enteroviruses and parechoviruses using RNA transcripts. J Med Virol. 2020;92(8):1065-74.

28. Tiveljung-Lindell A, Rotzén-Ostlund M, Gupta S, Ullstrand R, Grillner L, Zweygberg-Wirgart B, et al. Development and implementation of a molecular diagnostic platform for daily rapid detection of 15 respiratory viruses. J Med Virol. 2009;81(1):167-75.

.
